# Hospitalization in older adults: association with multimorbidity, primary health care and private health plan

**DOI:** 10.1590/S1518-8787.2017051006646

**Published:** 2017-04-24

**Authors:** Bruno Pereira Nunes, Mariangela Uhlmann Soares, Louriele Soares Wachs, Pâmela Moraes Volz, Mirelle de Oliveira Saes, Suele Manjourany Silva Duro, Elaine Thumé, Luiz Augusto Facchini

**Affiliations:** I Departamento de Enfermagem. Faculdade de Enfermagem. Universidade Federal de Pelotas. Pelotas, RS, Brasil; II Programa de Pós-Graduação em Enfermagem. Universidade Federal de Pelotas. Pelotas, RS, Brasil; III Faculdade Anhanguera de Pelotas. Pelotas, RS, Brasil; IV Departamento de Medicina Social. Faculdade de Medicina. Universidade Federal de Pelotas. Pelotas, RS, Brasil

**Keywords:** Aged, Comorbidity, Hospitalization, Prepaid health care plans, Family Health Strategy

## Abstract

**OBJECTIVE:**

Evaluate the association of multimorbidity, primary health care model and possession of a private health plan with hospitalization.

**METHODS:**

A population-based cross-sectional study with 1,593 elderly individuals (60 years old or older) living in the urban area of the city of Bagé, State of Rio Grande do Sul, Brazil. The outcome was hospitalization in the year preceding the interview. The multimorbidity was evaluated through two cut-off points (≥ 2 and ≥ 3). The primary health care model was defined by residence in areas covered by traditional care or by Family Health Strategy. The older adults mentioned the possession of a private health plan. We performed a gross and adjusted analysis by Poisson regression using a hierarchical model. The adjustment included demographic, socioeconomic, functional capacity disability and health services variables.

**RESULTS:**

The occurrence of overall and non-surgical hospitalization was 17.7% (95%CI 15.8–19.6) and 10.6% (95%CI 9.1–12.1), respectively. Older adults with multimorbidity were admitted to hospitals more often when to older adults without multimorbidity, regardless of the exhibition’ form of operation. Having a private health plan increased the hospitalization by 1.71 (95%CI 1.09–2.69) times among residents in the areas of the Family Health Strategy when compared to elderly residents in traditional areas without a private health plan.

**CONCLUSIONS:**

The multimorbidity increased the occurrence of hospitalizations, especially non-surgical ones. Hospitalization was more frequent in older adults with private health plan and those living in Family Health Strategy areas, regardless of the presence of multiple diseases.

## RESUMO

**OBJETIVO:**

Avaliar a associação da multimorbidade, modelo de atenção básica e posse de plano de saúde com hospitalização.

**MÉTODOS:**

Estudo transversal de base populacional com 1.593 idosos (60 anos ou mais) residentes na zona urbana do município de Bagé, Rio Grande do Sul. O desfecho foi a hospitalização no ano anterior à entrevista. A multimorbidade foi avaliada por meio de dois pontos de corte (≥ 2 e ≥ 3). O modelo de atenção básica foi definido pela residência em áreas cobertas pela atenção tradicional ou da Estratégia Saúde da Família. A posse de plano de saúde foi referida pelos idosos. Realizou-se análise bruta e ajustada por regressão de Poisson utilizando modelo hierarquizado. O ajuste incluiu variáveis demográficas, socioeconômicas, capacidades funcionais e de serviços de saúde.

**RESULTADOS:**

A ocorrência de hospitalização geral e não cirúrgica foi de 17,7% (IC95% 15,8–19,6) e 10,6% (IC95% 9,1–12,1), respectivamente. Idosos com multimorbidade hospitalizaram mais em comparação com os idosos sem multimorbidade, independentemente da forma de operacionalização da exposição. O plano de saúde aumentou em 1,71 (IC95% 1,09–2,69) vezes a internação hospitalar entre residentes nas áreas da Estratégia Saúde da Família em comparação aos idosos residentes nas áreas tradicionais sem plano de saúde.

**CONCLUSÕES:**

A multimorbidade aumentou a ocorrência de hospitalizações, principalmente aquelas não cirúrgicas. Idosos com plano de saúde e residentes em áreas de Estratégia Saúde da Família internaram mais, independentemente da presença de múltiplas doenças.

## INTRODUCTION

Hospitalization is an important resource in older adults care, and it is part of the health care network. Hospitalizations, especially if repeated and prolonged, may produce negative consequences to older patients’ health, such as decreased functional disability, lower quality of life and increased fragility^6^ ^,^ ^7^. Older adults hospitalization should be indicated only when all other actions and services for the proper management of health problems have been exhausted.

Health needs, mainly those expressed by chronic diseases, are one of the main determinants of elderly hospitalization^16^. With the rapid increase – absolute and relative – of the aging population, the prevalence of older adults with multiple chronic problems has reached 60%^11^. Therefore, there is a growing interest in elderly multidimensional assessment and in the implications triggered by multimorbidity for the organization and offer of health actions and services^20^ ^,^ ^26^.

In Brazil, the Ministry of Health adopted the Family Health Strategy (FHS) to reorganize primary health care^a^, reduce social inequalities and manage the care of older adults in the Brazilian Unified Health System (SUS)^7^. Through the work of a multidisciplinary team, the FHS is responsible for a population assigned to its territory so that it can act on health prevention and promotion, in addition to taking care of demand and provision of supplies. This care model has contributed to the reduction of infant mortality, hospitalizations by primary care-sensitive conditions^21^ and even cardiovascular deaths^24^. However, most of the evidence comes from ecological studies, which limits inference about the findings. Regarding the use of health services, findings with individual information show that FHS appears to offer a more suitable and equitable utilization of health services when compared to the traditional model^9^ ^,^ ^25^, but hospitalization related information are rarely found.

There are some studies being done to create instruments for hospitalization prediction in order to organize the work process of the primary health care teams^7^. The avoidable hospitalizations may represent a warning related to the lack of care coordination and the lack of care quality, possibly because of fragmentation when addressing the problems and especially in older adults with multimorbidity^8^ ^,^ ^19^ ^,^ ^26^. In this context, the possession of private health plans, regardless of the user’s needs, seems to be a facilitator of access to hospitalization in Brazil^23^, however, an assessment of the systems’ double coverage is seldom explored by the literature^17^.

Therefore, the objective was to evaluate the association of multimorbidity, primary health care model, and possession of a private health plan with senior hospitalization.

## METHODS

A population-based cross-sectional study with data collected between July and November 2008, including individuals 60 years old or older, living in the area covered by the primary health care services of the urban area of the city of Bagé, located on the border of Rio Grande do Sul State with Uruguay. In 2008, Bagé had about 120,000 inhabitants, of whom 84% lived in urban areas. Altogether, there were 20 primary care services, and 15 of them were part of the FHS and five belonged to the traditional model. The FHS had been in the city for five years and covered half of the urban population. The traditional model of primary health care was responsible for the care of the rest of the population. Older adults accounted for approximately 14% of the population. The city had three hospitals (an army hospital not affiliated with SUS) and 470 hospital beds affiliated with SUS (one bed per 3,800 inhabitants)^21^.

The sample size was calculated for a study on home care^25^. Considering 10% of losses and refusals, and a design effect of 1.3, the study had 80% power to detect 1.5 of relative risks and exhibitions that affected at least 4% of the population.

In the sample delimitation, the range of each primary health units was set and subsequently divided into micro areas, with the numeric ID of each block. The starting point of the data collection in each block was selected randomly and each home on the left was eligible, with one in six households being approached. We invited all residents 60 years old or older to participate in the study. The interviews not conducted after three attempts on different days and times were considered losses or refusals.

The interviews were carried out using structured questionnaires with pre-codified questions and applied by trained interviewers to all seniors in the home selected. In the event of partial disability – older adult with communication skills, lucid, oriented, but in need of daily care – family members or the main caregivers provided the answers. Self-reported questions were not applied in the case of total disability – elderly people incapable of communicating, completely reliant on family members or caregivers.

The outcome “hospitalization in the last year” was defined by the question: “Have you been admitted to any hospital in the last year? (yes; no)” In order to specify the outcome, we created a variable that included only non-surgical hospitalizations. The reason for the hospitalization was obtained through information mentioned by the interviewed.

The main independent variable were multimorbidity, primary health care models (traditional and FHS) and possession of a private private health plan. The multimorbidity was measured by 17 health problems: medical diagnosis mentioned by the older adults (systemic arterial hypertension, diabetes mellitus, pulmonary problems, heart disease, stroke, rheumatism/arthritis/osteoarthritis, spine problems, cancer, kidney failure); cognitive deficit evaluated by the Mini-Mental State Examination; Geriatric Depression Scale-evaluated depression; problems mentioned (urinary incontinence, amputation, visual or auditory problems, problem or difficulty chewing food, and falls)^22^. For the multimorbidity’s operation, we added up the health problems and used two categorizations: a) ≥ 2 morbidities; and b) ≥ 3 morbidities. The primary health care model was defined in the sample selection by residence in areas covered by health services^25^. Possession of a private health plan (no; yes) was mentioned by the older individuals.

The variables used to adjustment were: sex (male, female), self-reported skin color (white, black, yellow, brown or indigenous), age (60 to 64, 65 to 69, 70 to 74, 75 years or older), marital status (married or common-law, widowed, single or divorced), retirement (no, yes), years of study (none, one to seven, eight or more), economic classification according to the Brazilian Association of Research Companies (ABEP – A or B [richest]; C; D or E), functional disability for basic activities of daily living (AVD – no, yes)^13^, functional disability to instrumental activities of daily living (AIVD – no, yes)^15^, and home care in the three months preceding the interview (no, yes). For AVD and AIVD, seniors who reported needing help with at least one of the activities were considered disabled.

The analyses included calculations of ratios and their 95% confidence intervals. We performed a crude and adjusted analysis by Poisson regression with a robust adjustment in variance^2^. In the association between hospitalization and multimorbidity, we adjusted for gender, age, skin color, marital status, economic classification, and education. When associating primary health care model and private health plan, we included in the adjustment the disability variables for AVD and AIVD and home care. Saved the limitations of cross-sectional design, functional disabilities are the result of multimorbidity and can affect the use of home care^18^. The health services coverage (primary health care model and private health plan) can mediate or modify the association with hospitalization^17^. Thus, we tested the interaction between primary health care model and private health plan due to a theoretical suspicion of a relationship between these systems the outcome being studied^14^ ^,^ ^17^. Associations with a value of p ≤ 0.05 were considered statistically significant. Data analysis was performed using the program Stata, version 12.0 (StataCorp, CollegeStation, TX, USA).

The project was submitted to and approved by the Ethics Committee of the Faculdade de Medicina da Universidade Federal de Pelotas (Process 015/08). The ethical principles were assured. All respondents or their guardians signed consent forms.

## RESULTS

We interviewed 1,593 older adults. The study showed 4.0% of losses and 3.0% refusals. The prevalence of overall and non-surgical hospitalization was 17.7% (95%CI 15.8–19.6) and 10.6% (95%CI 9.1–12.1), respectively (Table 1). The main causes of hospitalization were: circulatory (30.4%), digestive (16.4%), general (13.3%), respiratory (10.7%), and urinary (7.2%). Of the total, 81.3% (95%CI 79.3–83.3) of the older adults had two or more morbidities and 64.0% (95%CI 61.5–66.4), had three or more morbidities. Half of the sample resided in areas covered by the FHS (Table 1).

**Table 1:** Description of the sample and multimorbidity and hospitalization prevalence in the elderly population. Bagé, State of Rio Grande do Sul, Brazil, 2008. (N = 1,593)

Variable	Sample		Multimorbidity (≥ 2)	Multimorbidity (≥ 3)	Hospitalization	Non-surgical hospitalization
				
	n	%	% (95%CI)	% (95%CI)	% (95%CI)	% (95%CI)
Gender						
Male	593	37.2	67.3 (63.4–71.3)	45.9 (41.7–50.1)	18.4 (15.3–21.5)	10.6 (8.1–13.1)
Female	1,000	62.8	82.1 (80.0–84.6)	65.3 (62.3–68.4)	17.3 (15.0–19.6)	10.6 (8.7–12.5)
Skin color						
White	1,252	78.6	74.7 (72.2–77.2)	55.1 (52.2–57.9)	18.2 (16.1–20.4)	10.4 (8.7–12.1)
Black	139	8.7	79.8 (72.7–86.9)	68.5 (60.3–76.8)	18.0 (11.6–24.4)	13.8 (8.0–19.5)
Brown/Yellow/Indigenous	202	12.7	86.2 (81.3–91.2)	70.4 (63.8–76.9)	14.4 (9.5–19.2)	10.1 (5.9–14.2)
Age (in completed years)						
60–64	400	25.1	72.4 (68.0–77.0)	52.0 (46.9–57.0)	14.3 (10.9–17.7)	8.4 (5.6–11.1)
65–69	374	23.5	72.1 (67.5–76.8)	55.4 (50.3–60.6)	15.8 (12.1–19.5)	7.5 (4.8–10.2)
70–74	322	20.2	77.7 (73.0–82.5)	57.8 (52.2–63.4)	19.9 (15.5–24.3)	10.7 (7.2–14.1)
≥ 75	497	31.2	83.3 (79.8–86.8)	66.1 (61.6–70.5)	20.5 (17.0–24.1)	14.8 (11.6–17.9)
Marital status						
Married or common-law	816	51.2	80.2 (77.4–83.0)	61.8 (58.4–65.2)	18.2 (15.5–20.8)	10.3 (8.2–12.4)
Single or divorced	238	15.0	79.3 (73.9–84.8)	60.6 (54.0–67.1)	16.8 (12.0–21.6)	10.2 (6.3–14.0)
Widowed	538	33.8	83.9 (80.6–87.1)	68.8 (64.7–72.9)	17.5 (14.3–20.7)	11.4 (8.7–14.1)
Retired						
No	451	28.3	81.9 (78.2–85.6)	65.5 (60.9–70.0)	15.1 (11.8–18.4)	9.1 (6.4–11.8)
Yes	1,142	71.7	81.1 (78.7–83.4)	63.4 (60.4–66.3)	18.8 (16.5–21.0)	11.2 (9.4–13.1)
Education (in complete years)						
None	372	23.7	87.1 (83.5–90.7)	72.1 (67.4–76.9)	16.9 (13.1–20.8)	11.4 (8.1–14.6)
1–7	858	54.5	76.9 (74.0–79.9)	57.3 (53.8–60.7)	18.7 (16.1–21.3)	10.3 (8.2–12.3)
≥ 8	342	21.8	64.8 (59.5–70.0)	45.3 (39.8–50.8)	16.7 (12.7–20.6)	10.7 (7.4–14.0)
Economic classification						
A or B (richest)	429	27.1	69.1 (64.6–73.7)	51.3 (46.3–56.2)	18.5 (14.8–22.1)	10.1 (7.2–13.0)
C	615	38.9	75.1 (71.6–78.7)	55.7 (51.6–59.8)	17.9 (14.9–20.9)	11.1 (8.6–13.6)
D or E	537	34.0	84.0 (80.8–87.2)	66.1 (62.0–70.3)	17.1 (13.9–20.3)	10.6 (7.9–13.2)
Functional disability for AVD						
No	1,424	89.4	80.1 (78.0–82.2)	61.8 (59.2–64.3)	15.5 (13.6–17.3)	8.5 (7.0–9.9)
Yes	169	10.6	96.3 (92.7–99.9)	91.7 (86.4–96.9)	36.7 (29.4–44.0)	29.3 (22.3–36.3)
Functional disability for AIVD						
No	1,045	65.8	76.8 (74.1–79.4)	55.7 (52.6–58.7)	13.0 (11.0–15.1)	6.5 (5.0–8.0)
Yes	544	34.2	91.7 (89.2–94.2)	82.9 (79.5–86.4)	26.8 (23.1–30.6)	18.8 (15.5–22.1)
Private health plan						
No	1,025	64.6	82.8 (80.4–85.2)	65.6 (62.6–68.7)	16.1 (13.9–18.4)	9.8 (7.9–11.6)
Yes	561	35.4	78.8 (75.3–82.2)	61.3 (57.1–65.4)	20.3 (17.0–23.7)	11.8 (9.1–14.4)
Primary health care model						
Traditional	741	46.5	77.9 (74.8–81.1)	59.7 (56.0–63.4)	17.3 (14.5–20.0)	9.6 (7.4–11.7)
FHS	852	53.5	84.2 (81.6–86.7)	67.6 (64.3–70.8)	18.1 (15.5–20.7)	11.5 (9.4–13.7)
Home care						
No	1,482	93.1	80.4 (78.3–82.4)	62.3 (59.7–64.8)	15.8 (13.9–17.7)	9.0 (7.5–10.5)
Yes	109	6.9	96.5 (92.5–100)	90.6 (84.3–96.8)	43.1 (33.8–52.5)	32.4 (23.4–41.4)
Total	1,593	100	81.3 (79.3–83.3)	64.0 (61.5–66.4)	17.7 (15.8–19.6)	10.6 (9.1–12.1)

AVD: activities of daily living; DLIA: instrumental activities of daily living; FHS: Family Health Strategy

Almost 2/3 of the sample were women. White skin color was the most mentioned (78.6%). Older adults aged between 60 and 64 years old accounted for 25.1% and those with 75 years or older comprised 31.2% of individuals. Half were married or lived with a partner and 33.8% were widowers. Two-thirds were retirees and most older adults had between one and seven years of education (54.5%) and 23.7% of them had never attended school. The economic classes D/E and C were composed of 34.0% and 38.9% of the older adults, respectively. The occurrence of AVD was 10.6% and 34.2% for AIVD. One-third had a private health plan and 6.9% received home care. The occurrence of hospitalization – overall and non-surgical – was higher among individuals with AVD, with AIVD or receiving home care. The occurrence of hospitalization was similar between the categories of other variables investigated (Table 1).

**Figure 1:**
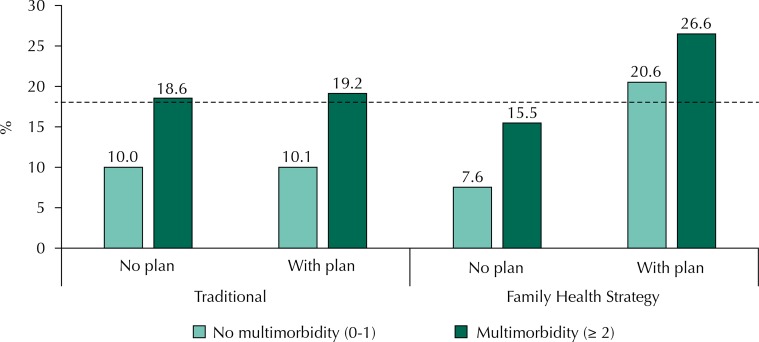
Prevalence of hospitalization according to the presence of multimorbidity stratified by primary care model and private health plan in the elderly population. Bagé, State of Rio Grande do Sul, Brazil, 2008.

There was an interaction between the model of primary health care and private health plan, both in the crude analysis (p = 0.004) and adjusted analysis (p = 0.020). In stratified analysis, the occurrence of hospitalization was greater among elderly residents in areas covered by the FHS and with the private health plan, regardless of the presence of multimorbidity (Figure 1).

Dotted line: overall hospitalization %

Hospitalizations were more frequent in older adults with multimorbidity. The magnitude of the association was greater for multimorbidity defined by three or more diseases and for nonsurgical hospitalization. On the coverage of health services, in the adjusted analysis, residents in areas covered by the FHS with private health plan had 1.71 (95%CI 1.09–2.69) times more of overall admission and 2.20 (95%CI 1.16–4.19) times more non-surgical hospitalization when compared to seniors living in areas covered by the traditional care model and no health insurance (Table 2).

**Table 2:** Association between hospitalization, multimorbidity, and health services coverage among older adults. Bagé, State of Rio Grande do Sul, Brazil, 2008. (N = 1,593)

Variable	Hospitalization		Non-surgical hospitalization	
	
	Gross analysis	Adjusted analysis^c^	Gross analysis	Adjusted analysis^c^
			
	PR (95%CI)	PR (95%CI)	PR (95%CI)	PR (95%CI)
Multimorbidity (≥ 2)^a^	p = 0.003	p = 0.003	p = 0.003	p = 0.003
No	1	1	1	1
Yes	1.73 (1.21–2.47)	1.75 (1.21–2.51)	2.35 (1.35–4.09)	2.34 (1.34–4.08)
Multimorbidity (≥ 3)^b^	p < 0.001	p < 0.001	p < 0.001	p < 0.001
No	1	1	1	1
Yes	1.83 (1.40–2.40)	1.94 (1.46–2.56)	2.87 (1.88–4.39)	3.10 (2.01–4.80)
Primary health care model/Possession of a private health plan	p = 0.002	p = 0.011	p = 0.002	p = 0.021
Traditional/No plan	1	1	1	1
Traditional/With plan	0.92 (0.67–1.27)	1.01 (0.71–1.42)	0.74 (0.46–1.17)	0.84 (0.50–1.39)
FHS/Without plan	0.84 (0.64–1.12)	0.80 (0.58–1.09)	0.85 (0.59–1.24)	0.80 (0.51–1.21)
FHS/With plan	1.89 (1.23–2.91)	1.68 (1.07–2.64)	2.60 (1.43–4.74)	2.20 (1.16–4.19)

PR: prevalence ratio

Reference groups: ^a^ 0-1 disease; ^b^ 0-2 diseases; ^c^ adjusted analysis – multimorbidity: adjustment for demographic and socioeconomic variables; primary health care model/possession of a private health plan: adjustment for sociodemographic, socioeconomic, multimorbidity, functional disabilities and home care variables.

Individuals residing in the areas covered by FHS were poorer, less educated, and had more limitations in ADL and AIVD compared to residents in the traditional model. Possession of a private health plan was 26.7% in the areas covered by FHS, and 45.3% in the traditional model. When stratifying older adults by the model of primary care and private health plan, we observed that richer and more educated older adults resided in traditional areas with the private health plan, followed by residents in areas covered by the FHS with a private health plan (Table 3).

**Table 3:** Description (%) of socioeconomic characteristics and of the health condition according to the primary health care model and private health plan in the elderly population. Bagé, State of Rio Grande do Sul, Brazil, 2008.

Socioeconomic and health condition variables	Traditional		FHS	
	
	No plan	With plan	No plan	With plan
Economic classification				
A or B (richest)	21.8	54.1	10.2	43.6
C	42.4	36.6	37.9	38.7
D or E	35.7	9.3	51.9	17.8
Education (in complete years)				
None	19.4	9.6	36.1	19.0
1–7	60.0	44.2	56.5	55.3
≥ 8	20.6	46.2	7.5	25.7
Multimorbidity (≥ 2)	80.8	74.8	84.0	84.4
Multimorbidity (≥ 3)	63.6	55.4	67.0	69.7
AVD	8.4	9.9	12.2	11.1
AIVD	29.7	28.4	39.0	37.8

FHS: Family Health Strategy; AVD: basic activities of daily living; AIVD: instrumental activities of daily living

## DISCUSSION

The multimorbidity increased the occurrence of hospitalizations. Older adults with private health plans and those living in Family Health Strategy areas were admitted more often, regardless of the presence of multiple diseases. Considering the relevance of FHS in organizing the network and coordination of care, the public-private relationships and the impact of chronic diseases in the health system^19^, this is one of the first Brazilian studies with primary data to evaluate the effect of multimorbidity on hospitalization and the role of health services coverage. Most of the existing information in the current literature comes from ecological studies.

The positive association between multiple diseases in the same individual and hospitalization is consistent with literature findings^16^. It is explained by the increased risk of physiological complications arising from diseases. The comparison of the results with different cut-off points for multimorbidity showed that the use of three or more diseases was a richer indicator when predicting hospitalization, reinforcing the use of this cut-off point in the evaluation of multimorbidity in older adults^12^. Nevertheless, these results represent important challenges for elderly care in Brazil, because of the higher the number of diseases, the greater the likelihood of hospitalization. Due to its negative consequences^6^, hospitalization should be used, reinforcing the proper management of multimorbidity in primary health care^a^ and including the residence as a therapeutic environment^19^ ^,^ ^20^ ^,^ ^25^.

Despite the theoretical framework, the actions and services provided by the FHS and the existing of evidence that suggests a care of better quality for the FHS’s chronic problems^19^, this study did not find an isolated effect of this model of care in the association between multimorbidity and hospitalization when compared with the traditional model. This result raises some hypotheses. In the city of study, in 2008, the FHS was not consolidated enough to influence the reduction in elderly hospitalization. It is necessary to emphasize that the implementation of the FHS was being carried out gradually in the city and, on average, the primary health units with FHS had three years of deployment.

In addition, the sources for the literature’s findings indicating the FHS’s positive effect are mostly ecological evidence, which limits causal inference^21^ ^,^ ^23^ ^,^ ^24^. Both studies with individual data, hospital-based and no age restriction showed different results for the hospitalizations due to conditions sensitive to primary health care despite the different forms of assessing the exposure to the FHS. A study performed in the same city as this study found no differences in the likelihood of hospitalizations for primary care-sensitive conditions among the models of care^21^. On the other hand, a study in a city from another region of the country detected a lower ratio of hospitalization for primary care-sensitive conditions among individuals connected to the FHS^10^. Finally, some features of this study may justify the results observed. The FHS assessment was based on residence in the areas of service and not on the connection and regular use of services with this care model. Despite the accountability of the FHS teams by a population assigned to the territory^a^, this form of operation may have diluted a possible model, because some older adults may not use the FHS service.

Corroborating with the findings of this study, national^3^ ^,^ ^5^ and international^27^ literature highlights the increase in utilization of services by individuals with a private health plan. However, the plausibility of the findings on the private health plan’s effect on the increase in hospitalization in FHS areas is complex and involves from socio-economic and health condition characteristics up to aspects related to access to hospitalization. Older adults in FHS areas were poorer, less educated, had more functional disabilities and more multimorbidity, which would stimulate a greater demand for care. However, only those who had a private health plan were admitted more often, probably by having easier access to hospitalization^23^, even if the management of a health problem could be performed at home or in outpatient service. In addition, in Brazil, the difficulties in supply and provision of beds in the public health system induce a greater occurrence of hospitalization in the supplementary system, mostly for hospitalization of lesser complexity and lower duration^1^. The hospitalizations paid for by a private health plan went up from 6% in 1981 to 20% in 2008. Meanwhile, the public system financed hospitalizations decreased, and those paid for by direct disbursement remained stable. Still, we must consider the medical option when using a private health plan to accelerate access to diagnostic and therapeutic resources in treatment^10^, especially among people with less purchasing power^21^. The assessment of the private health plan’s role as a facilitator of hospitalization should be deepened, with a focus on the user’s needs and an assessment of the quality provided by the supplementary system.

Despite the small isolated influence of the services in changing the population’s health conditions^4^, multimorbidity can be one of the main focuses for effective action of health systems and services in the short term. Taking into consideration the large population of older adults affected by the problem and the low probability of a quick reduction in multimorbidity, actions must focus on the tertiary and quaternary prevention in order to prevent complications of multiple diseases, iatrogenic and unnecessary use of health services^20^.

Some limitations of the study must be balanced. The cross-sectional design did not allow inferences on the risk of hospitalization, since the hospitalization may have increased the chance of disease diagnosis. The lack of specificity in outcomes that did not distinguish hospitalizations for primary care-sensitive conditions may have hampered the adequate assessment of the association with the care model. In addition, due to the lack of information on financing and access to hospitalization, it was not possible to obtain more details about the trajectory of access to hospitalization, especially among the FHS’s older adults – poorer and the main unique users of the public health system^23^.

The lack of identification of the FHS’s effect on the association between multimorbidity and hospitalization reinforces the need for future studies at an individual level, with longitudinal information that also includes questions about the lack of access to hospitalization, the effective connection with the FHS and hospitalizations for primary care-sensitive conditions. The findings indicate the relevance of multimorbidity in the event of hospitalization and can contribute to improvements in the care model for older adults in Brazil.

## Footnotes

a Ministério da Saúde (BR), Secretaria de Atenção à Saúde, Departamento de Atenção Básica. Política Nacional de Atenção Básica. Brasília (DF); 2012 [cited 2017 Feb 15]. (Série E. Legislação em Saúde). Available from: http://189.28.128.100/dab/docs/publicacoes/geral/pnab.pdf
